# Epidemiology of COVID-19 in Individuals under 18 Years Old in Cartagena, Colombia: An Ecological Study of the First 14 Months of the Pandemic

**DOI:** 10.3390/tropicalmed7060107

**Published:** 2022-06-15

**Authors:** Steev Loyola, Eder Cano-Pérez, Jaison Torres-Pacheco, Dacia Malambo-Garcia, Ruben Gomez, Doris Gomez-Camargo

**Affiliations:** 1 Molecular Research Unit (UNIMOL), Faculty of Medicine, University of Cartagena, Cartagena de Indias 130014, Colombia; ecanop@unicartagena.edu.co (E.C.-P.); jtorresp@unicartagena.edu.co (J.T.-P.); dmalambog@unicartagena.edu.co (D.M.-G.); 2 PhD Program in Tropical Medicine, Faculty of Medicine, University of Cartagena, Cartagena de Indias 130014, Colombia; rdgomez@ces.edu.co; 3 Faculty of Medicine, Universidad Peruana Cayetano Heredia, Lima 150135, Peru

**Keywords:** SARS-CoV-2, COVID-19, child, adolescent, Colombia

## Abstract

The epidemiology of the coronavirus disease (COVID-19) has been scarcely described in individuals under 18 years old, particularly during the first months of the pandemic. The study aimed to describe the COVID-19 epidemiology in the Colombian department of Bolívar from March 2020 to April 2021 among individuals under 18 years. Furthermore, we explored whether the use of data generated by a Bolívar reference laboratory captures the departmental epidemiology. Two information sources were used; the national COVID-19 surveillance system and the Bolívar COVID-19 reference laboratory. In using a population-based ecological approach and information from confirmed symptomatic cases, epidemic curves and heat maps were constructed to assess the COVID-19 dynamics and patterns by sex, age, and residence (Cartagena vs. 45 other municipalities). The COVID-19 incidence was comparable between males and females but varied by age group, being higher in children aged 10 years and older. Cartagena had a significantly higher number of cases and experienced early epidemic peaks. Our analyses suggest that information generated by the reference laboratory does not capture the COVID-19 departmental epidemiology, despite conducting population-based surveillance across Bolívar. The study provides a retrospective characterization of the COVID-19 epidemiology in an understudied population and information that may be useful for future evidence-based responses.

## 1. Introduction

The severe acute respiratory syndrome coronavirus 2 (SARS-CoV-2) emerged in China in late 2019, and it is the causative agent of coronavirus disease (COVID-19) [1,2]. The World Health Organization, given the rapid spread of SARS-CoV-2 and the increase of COVID-19 cases globally, declared COVID-19 as a pandemic in mid-March 2020 [1]. As of late April 2022, more than 500 million confirmed cases and more than 6 million COVID-19-associated deaths have been reported worldwide [3]. In Colombia, the first officially COVID-19 case was reported in early March 2020; two years later, more than 6 million cases and more than 139,000 deaths have been reported [3,4]. In the Colombian Caribbean region, Bolívar was one of the most affected departments by the COVID-19 pandemic [4,5,6,7].

The epidemiology of COVID-19 has been largely described in adults but scarcely in individuals under 18 years old (infants, children, and adolescents) [8,9,10,11]. Studies conducted in several locations during the first months of the pandemic, including Colombia, suggested these individuals had a low infection rate in comparison to adults and that infections in infants and children were most likely to be associated with adult household contacts [7,8,12,13,14,15,16,17]. Recent investigations suggested that individuals under 18 still represent a minor proportion of cases in the population and that severe disease and death in children remain infrequent outcomes [8,9,14,17,18,19]. Immunosuppressed or immunocompromised children and those with transplants, cancer, congenital heart disease, rare diseases, or malnutrition are particularly prone to develop a severe illness or have a multisystem inflammatory syndrome, requiring hospitalization or admission to the intensive care unit [8,10,15,17,20]. In the absence of comorbidities and factors associated with severity, most infants, children, and adolescents with COVID-19 are asymptomatic or are likely to be mildly symptomatic [7,8,9,10,13,14].

The COVID-19 pandemic has heavily affected the well-being of individuals under 18. The closure of educational institutions constrained social interactions and development opportunities [9,12]. In Colombia, in late March 2020, early childhood education centers and schools were closed as part of the COVID-19-related non-pharmaceutical interventions (NPI) measures [6]; therefore, in-person learning was disrupted. In addition, the pandemic generated barriers to accessing mass immunization programs, given the reduced access to and provision of non-essential health services [9,21,22]. Specifically, the vaccination coverage against preventable diseases was significantly diminished during the first pandemic year in Colombia, affecting younger children and rural areas more severely [21]. Additionally, given the lack of information on safety and effectiveness, COVID-19 vaccination programs have included children and adolescents belatedly [9,10].

Infants, children, and adolescents are susceptible to and can transmit SARS-CoV-2; however, their role in the transmission dynamics during the first pandemic year remains poorly understood [9,20,23,24,25]. Furthermore, the COVID-19 burden in individuals under 18 has probably been underestimated as over 90% of cases do not develop severity or are asymptomatic [8,9,10,13,17,23]. The lack of an epidemiological comprehensive characterization hinders the implementation and redesign of evidence-based public health interventions and policies [26]. Hence, in circumstances where an evidence-based response can mitigate the impact and inform public health policies, the retrospective use of surveillance-derived data allows the characterization of COVID-19 dynamics and identification of priority groups [5,26]. Therefore, the aim of this study was to describe the epidemiology of COVID-19 in the department of Bolívar, Colombia, from the first officially registered case of COVID-19 in March 2020 to April 2021 in individuals under 18 years of age. Additionally, we explored whether the use of data generated by a reference laboratory that tests SARS-CoV-2 in suspected cases from Bolívar is sufficiently sensitive to capture departmental dynamics.

## 2. Materials and Methods

### 2.1. Study Design

This is a population-based ecological study that describes the epidemiology of COVID-19 cases in individuals under 18. All laboratory-confirmed cases reported for the department of Bolívar between 1 March 2020 and 30 April 2021 were included in the analysis. In late March 2020, the Colombian government issued several NPI to contain the SARS-CoV-2, including mask use, social distancing policies, mandatory preventive isolation, and closure of non-essential services and establishments [6]. Thereafter, from May 2020 onwards, NPI related to the mobilization were progressively relaxed to allow selective and gradual mobilization of the population [6]. Social, recreational, and educational activities for individuals under 18 remained restricted throughout the study period, and children under two years old were not required to wear masks. The Colombian Government’s COVID-19 vaccination program started in February 2021 and specifically targeted health personnel and adults over 80 years old [27,28]. Therefore, for the study period, individuals under 18 years did not have access to COVID-19 vaccines, and the emergence of SARS-CoV-2 variants was poorly understood.

The department of Bolívar is located in the Caribbean region of Colombia, and by 2020 had an estimated population of 2,180,976 inhabitants distributed heterogeneously in its 46 municipalities [29,30]. Cartagena de Indias (10°24′0″ N, 75° 30′0″ W) is the capital of the department of Bolívar, is an urban municipality, and by 2020 concentrated 47.2% of the department’s population [29]. Cartagena has an airport and seaports with domestic and international connections for commercial, industrial, and tourist activities. Consequently, Cartagena is considered one of the most important cities in the Caribbean region. The other 45 municipalities are predominantly rural, and their inhabitants are primarily involved in agriculture, fishing, and local trade [29,30].

### 2.2. Data Sources

Two information sources were used in this study. The records of the Unidad de Investigación Molecular (UNIMOL) of the Universidad de Cartagena constituted the first source of information. UNIMOL was the first laboratory licensed by the Colombian National Institute of Health (CNIH) to conduct SARS-CoV-2 screening in the department of Bolívar; thus, UNIMOL became a reference laboratory. Therefore, UNIMOL, in collaboration with the regional Public Health Laboratory (PHL) of Bolívar, has played a key role in the SARS-CoV-2 testing since March 2020. Nasopharyngeal swabs are routinely collected from suspected cases by health care institutions located throughout Bolívar, and then specimens are transported at a cold chain to the PHL located in Cartagena [31]. Subsequently, specimens are delivered to UNIMOL for testing SARS-CoV-2 viral RNA using a CNIH-approved RT-PCR [32]. The RNA extraction techniques and molecular methods used for viral RNA detection at UNIMOL were described elsewhere [33]. All confirmed cases by UNIMOL are routinely reported to the national COVID-19 surveillance system (SisMuestras COVID-19). Eventually, and given the growing necessity to scale up and massify the screening, several new CNIH-licensed laboratories started COVID-19 diagnostic activities in the department of Bolívar. For this reason, the second source of information relied on the available data recorded in the national COVID-19 surveillance system (https://www.datos.gov.co/Salud-y-Protecci-n-Social/Casos-positivos-de-COVID-19-en-Colombia/gt2j-8ykr/data (accessed on 18 June 2021)). The surveillance system is administered by the CNIH.

In Colombia, the suspected case definition was composed of epidemiological and clinical criteria [34]. At the beginning of the pandemic, individuals with a history of travel to or residence in a territory with documented transmission during the 14 days prior to symptom onset and with acute respiratory illness (fever, cough or shortness of breath) were suspected of having COVID-19. Over time, the epidemiological criterion changed to unprotected or sustained close contact exposure to probable or confirmed COVID-19 cases, while the clinical criterion remained virtually the same. Suspected cases of COVID-19 were routinely indicated for diagnostic testing, and cases with a positive result for SARS-CoV-2 were classified as confirmed COVID-19 cases [34]. Confirmed symptomatic cases accounted for more than 95% of the total records in both sources of information. For this study, the information of all laboratory-confirmed symptomatic cases diagnosed up to 30 April 2021 was downloaded from the two data sources and then analyzed.

### 2.3. Statistical Analysis

Sex, age, and place of residence were summarized by the information source. The estimated number of inhabitants under 18 for 2020 by the municipality, by sex, and by age was obtained from the Departamento Administrativo Nacional de Estadística of Colombia [29]. The cumulative incidence per 100,000 and its 95% confidence interval (95% CI) were estimated using the number of confirmed cases by the CNIH and the estimated population in 2020. The binomial exact method was used to estimate 95% CI. As Cartagena had a high rate of urbanization and domestic and international connections, it was analyzed separately, whereas the other 45 municipalities were analyzed on an aggregate basis. The frequency of COVID-19 cases, both corrected and non-corrected for population size, was evaluated using epidemic curves considering the date of diagnosis, date of symptom onset, and date of reporting using 7-day moving averages. Additionally, heat maps were constructed using population-corrected and log-transformed [log(x + 1)] COVID-19 cases. Age- and sex-stratified analyses were performed using symptom onset dates since these are not affected by delays in diagnosis or by logistical or administrative factors [35]. Data were analyzed in Stata v16 (StataCorp. 2019. Stata Statistical Software: Release 16. College Station, College Station, TX, USA: StataCorp LLC).

## 3. Results

Between March 2020 and April 2021, UNIMOL and CNIH reported a total of 1007 and 7673 individuals under 18 years who tested positive for SARS-CoV-2, respectively. Of the total cases registered on the national COVID-19 surveillance system, UNIMOL reported 13.1% (1007/7673) of the COVID-19 cases. Overall, the distribution of cases according to sex and age was similar to the information provided by UNIMOL and CNIH (Table 1). Regardless of the source of information, the frequency of COVID-19 cases was higher in Cartagena (Table 1).

**Figure 1:**
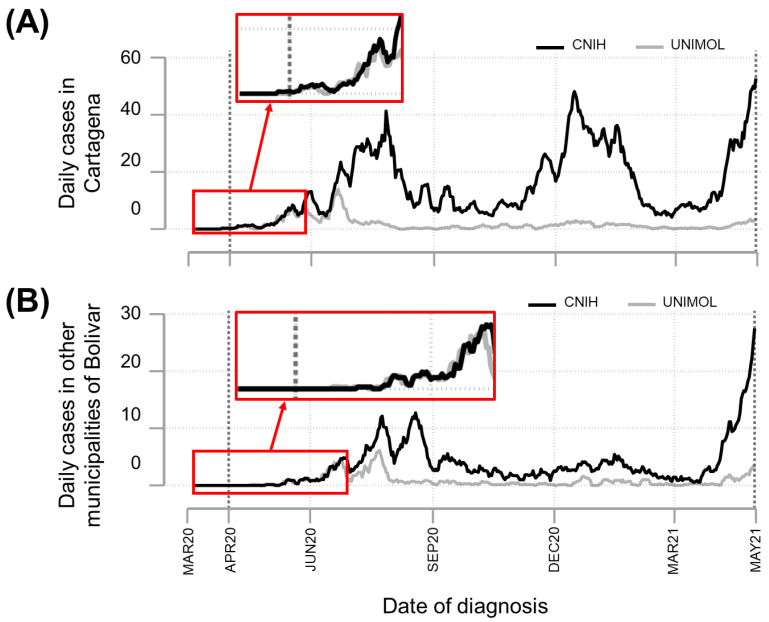
Comparison of epidemic curves based on data from two data sources, as of 31 April 2021. Daily new confirmed COVID-19 cases in Bolívar were used to build epidemic curves using 7-day moving averages. The curves were constructed using public information from the Colombian National Institute of Health (CNIH) and Unidad de Investigación molecular (UNIMOL) of the Universidad de Cartagena. Red boxes indicate the time frames for which the epidemic curves had a similar pattern; ~3 and ~4 months for Cartagena (**A**) and other Bolívar municipalities (**B**), respectively.

Assessing the epidemic curve by date of diagnosis and using the cases registered in the national surveillance system as a reference, the information provided by UNIMOL reflected the epidemiology of Cartagena and other municipalities in Bolívar for approximately the first three and four months, respectively (Figure 1). Given the lack of sensitivity of the information from the UNIMOL reference laboratory to detect epidemic peaks throughout the study period, subsequent analyses were performed using the information from the national surveillance system. Therefore, the characterization of the COVID-19 epidemiology presented here was conducted using the information provided by the national surveillance system.

The cumulative incidence of COVID-19 was comparable between males and females, whereas the incidence varied by age. Specifically, the observed incidence in children under one year (731.4, 95% CI: 649.6–820.5) and those from one to under five years (649.9, 95% CI: 654.8–736.9) were comparable and were also the lowest compared to the incidence observed in older individuals (Table 1). The highest incidence among the evaluated age groups was observed in the group of 10 to <14 years old (1610.2, 95% CI: 1548.0–1674.2, Table 1). Using information derived from the national COVID-19 surveillance system, the cumulative incidence of COVID-19 in Cartagena was 5.5× times the incidence observed in other municipalities of Bolívar.

**Figure 2:**
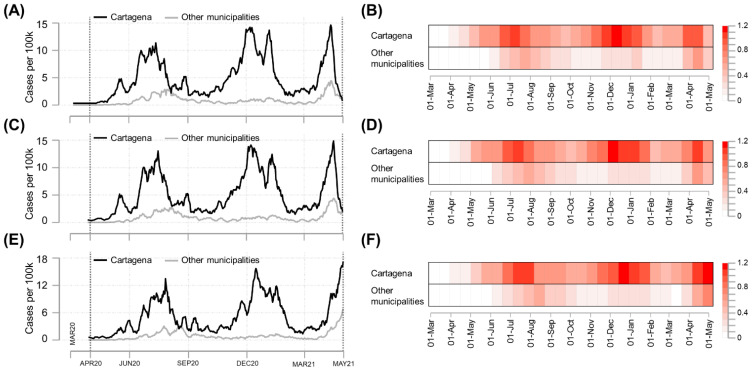
Epidemic curves and their patterns of daily new confirmed COVID-19 cases in Cartagena and other municipalities of Bolívar, as of 31 April 2021. Daily cases per 100,000 population were plotted using 7-day moving averages by date of symptom onset (panels (**A**,**B**)), date of reporting (panels (**C**,**D**)), and date of diagnosis (panels (**E**,**F**)). Epidemic curves (panels (**A**,**C**,**E**)) and heat maps (panels (**B**,**D**,**F**)) were constructed using linear and “log (x + 1)” scales to improve readability, respectively.

The epidemic curves according to symptom onset (Figure 2A) and reporting dates (Figure 2C) had a similar pattern for both Cartagena and other Bolívar municipalities. The epidemic curves by dates of diagnosis (Figure 2E) shifted to the right and exhibited a slightly different pattern compared to curves constructed using symptom onset and reporting dates. Interestingly, despite the date used for the analysis, heat maps suggested that peaks detected in Cartagena preceded those observed in the other municipalities of Bolívar (Figure 2B,D,F).

**Figure 3:**
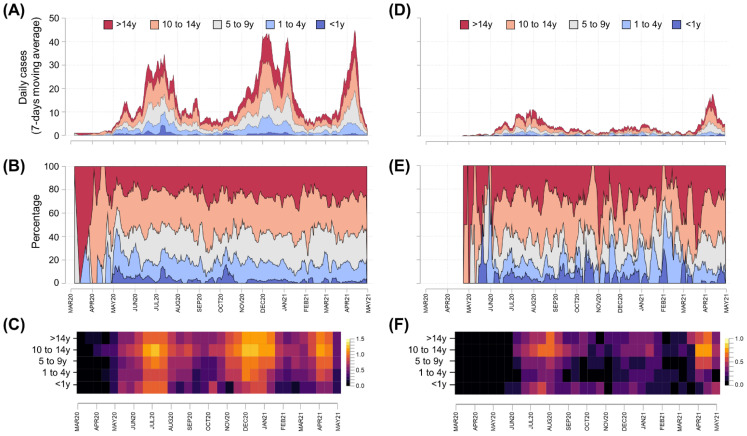
Age-stratified epidemic curves for Bolívar and Cartagena, as of 31 April 2021. Daily confirmed new cases were presented using epidemic curves (panels (**A**,**D**)) and stacked area plots (panels (**B**,**E**)) on linear scales, while cases per 100,000 population were presented using heat maps (panels (**C**,**F**)) with log (x + 1) scales to improve readability. Data for Cartagena (panels (**A**–**C**)) and other municipalities of Bolívar (panels (**D**–**F**)) were presented using symptom onset dates and 7-day moving averages.

**Figure 4:**
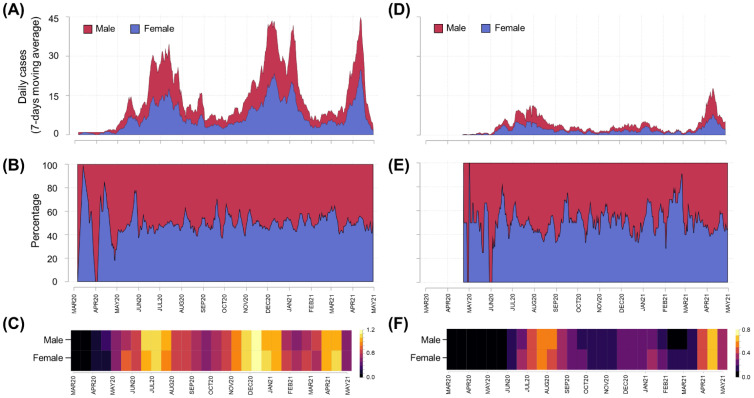
Sex-stratified epidemic curves for Bolívar and Cartagena, as of 31 April 2021. Daily confirmed new cases were presented using epidemic curves (panels (**A**,**D**)) and stacked area plots (panels (**B**,**E**)) on linear scales, while cases per 100,000 population were presented using heat maps (panels (**C**,**F**)) with log(x + 1) scales to improve readability. Data for Cartagena (panels (**A**–**C**)) and other municipalities of Bolívar (panels (**D**–**F**)) were presented using symptom onset dates and 7-day moving averages.

The case dynamics by age group and date of symptom onset were summarized for Cartagena and other municipalities in Bolívar (Figure 3). In comparison to other Bolívar municipalities, Cartagena had a larger number of cases (4.2×, Table 1) and major epidemic peaks between December 2020 and January 2021 (Figure 3A compared to D) that were apparently not related to outbreaks in specific age groups. The age group-specific incidence across the study period displayed low variation in Cartagena (Figure 3B,C), while a greater variability was observed in other Bolívar municipalities (Figure 3E,F). No difference was observed in the age-stratified occurrence (Figure 4A,C,D,F) or proportion (Figure 4B,E) of cases for Cartagena and the other municipalities of Bolívar.

**Figure 5:**
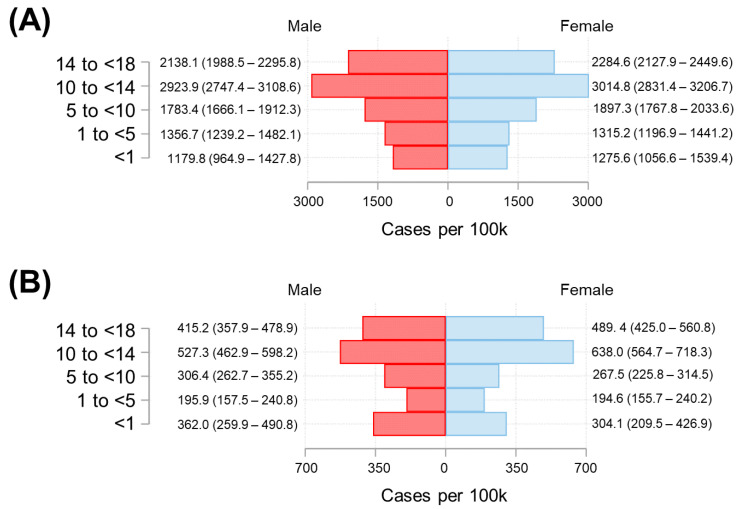
Distribution of cases by gender and age, as of 31 April 2021. The distribution of confirmed new cases in Cartagena and other municipalities of Bolívar per 100,000 population was plotted in Panel (**A**,**B**), respectively. The 95% confidence intervals of the cumulative incidences were in parenthesis.

The COVID-19 incidence among males and females was comparable after stratifying by age and residence (Figure 5). However, both in Cartagena (Figure 5A) and in other municipalities (Figure 5B), the incidence was higher in the 10 to <14 years old group compared to other age groups. Additionally, in Cartagena, the group with the lowest incidence of COVID-19 was children under one year of age, while in the other municipalities of Bolívar, the group with the lowest incidence was those aged 1 to <5 years of age.

## 4. Discussion

The transmission patterns and epidemiology of COVID-19 in individuals under 18 years old have been sparsely documented [8,9,11,17]. This lack of information hinders efforts to understand the underlying dynamics between epidemic peaks and the burden of COVID-19 [26]. In addition, insufficient information prevents prioritization for vaccination or booster vaccination of population subgroups at increased risk of severe disease or long-term COVID-19 complications among infants, children, and adolescents [5,9]. To our knowledge, this is the most comprehensive evaluation of the dynamics and epidemiology of COVID-19 in the Colombian department of Bolívar among individuals under 18 years, from the first reported case in March 2020 to April 2021.

The testing capacity has been crucial for the rapid and opportune investigation of COVID-19 cases as part of the various pandemic response and control activities [36]. Regarding the exploratory aim, our findings suggest that the information reported by the reference laboratory UNIMOL reflected the early dynamics of COVID-19 for Cartagena and other municipalities in Bolívar. Interestingly, although UNIMOL collaborated continuously with the PHL for the SARS-CoV-2 community-based testing in suspected cases throughout Bolívar, the information generated by UNIMOL was not sensitive enough to detect the increase of COVID-19 cases in Cartagena between July and September 2020, and November and February 2020, as well as the increase between August and September 2020 for other Bolívar municipalities (Figure 1). The reduced sensitivity to detect epidemic peaks was most likely related to the scale-up and increased diagnostic capacity as a result of the licensing of several laboratories for SARS-CoV-2 screening, which led, especially in Cartagena, to a significant increase in cases reported to the national COVID-19 surveillance system. Therefore, our results strongly suggest that the use of data generated by one laboratory may not be sufficient to capture and infer population dynamics despite having department-level diagnostic coverage.

COVID-19 has impacted urban and rural populations differently. Previous studies suggest that spatial and temporal COVID-19 patterns differed by multiple factors such as urbanization, pre-existing social and economic vulnerability, contact-type distributions, age group distribution, and the promptness of implementation and compliance with NPI [14,37,38,39]. In addition, it was previously suggested that cities with high population density and high mobility rates, as well as those with airports and international borders, played a key role in the entry and spread of SARS-CoV-2 to nearby locations [7,40,41,42]. An initial description of the epidemiology of COVID-19 in the municipalities of Bolívar suggested that burden and mortality were significantly higher in Cartagena compared to other municipalities [33]. Here, we evaluated the epidemiological patterns of COVID-19 in Cartagena and other municipalities to assess the progression of the pandemic using information from the national surveillance system. We observed that, regardless of the date used for the analysis, the epidemic peaks in Cartagena consistently preceded those that occurred in other Bolívar municipalities. Based on previous reports describing the role of urbanized cities over rural areas [39,40,42], we hypothesized that Cartagena acted as a “super-spreader city” and played a key role in the entry and spread of SARS-CoV-2 to Bolívar rural localities. However, taking into account the analyzed data, we certainly cannot rule out the hypothesis that the viral entry and spread also occurred from other nearby localities (such as capital cities of nearby departments other than Bolívar) and not exclusively from Cartagena. In rural areas, especially in low-resource settings, delayed epidemic peaks could be related to typical rural population characteristics such as population dispersion and reduced contact rates. Furthermore, the reduced number of cases could be related to the difficulty in improving or maintaining a high diagnostic capacity in resource-limited settings [12].

The incidence of COVID-19 was comparable between males and females throughout the study period for both Cartagena and other Bolívar municipalities, using information from the national surveillance system. These findings concur with those previously described for other countries and other Colombian regions [7,23,24]. The age-stratified incidence of COVID-19 varied slightly throughout the study period for Cartagena, while in other municipalities of Bolívar, the variation was greater; however, the group with the highest incidence was individuals with 10 or more years of age in both Cartagena and other municipalities. The observed low incidence in younger children could be explained by reduced exposure to older individuals, as well as by an under-representation of younger age groups, given that younger children are more likely to be asymptomatic [8,13,43]. Moreover, it is conceivable that the low incidence observed in younger children could be related to infrequent testing in this group since they are more likely to have mild symptoms or asymptomatic disease compared to older children [23]. However, as we used data from symptomatic cases and given the high probability of being asymptomatic, our findings very likely underestimate the COVID-19 burden among individuals younger than 18 years of age.

This ecological study is subject to multiple limitations. First, we did not assess clusters or spatiotemporal patterns at lower geographic scales (such as neighborhoods) because the information was not available. A previous study conducted in a Colombian city in the Caribbean region suggested that COVID-19 epidemic peaks were concentrated in specific areas of the city [14]. Thus, the COVID-19 epidemiology described for Cartagena and other municipalities could not reflect dynamics and patterns at lower scales. Furthermore, aggregate information from multiple municipalities in one category could not reflect the epidemiology of each locality even though they have similar rural characteristics. Second, we failed to evaluate changes in the COVID-19 dynamics and patterns by potential confounders or effect modifiers. Infrastructure, coverage, strengthening of local health services, and socioeconomic and demographic characteristics have been described as socio-ecological factors that influence the epidemiology of COVID-19 [14,26]. Similarly, underlying medical conditions, antibody waning, and reinfection are individual-level factors that influence the incidence of COVID-19 among children and adolescents [14,15,26]. Further studies are needed to better understand the impact of socio-ecological and individual factors on the epidemiology of COVID-19 among children under 18 years. Third, mobility and age-structured contact rates were not incorporated into the analysis. Mobility and contact rates have been described as time-varying factors that influence the epidemiology of COVID-19, including in Colombia [26,44,45]. As of mid-May 2020, mobilization restrictions were relaxed for adults across Colombia [6], so it is plausible to assume that adults with higher SARS-CoV-2 exposure in the community posed a risk to children and other co-resident members [9,24]. Hence, it is also reasonable to assume that most of the cases registered during the first months of the pandemic were secondary household infections and that the high incidence of COVID-19 in individuals aged 10 and older could be related to increased contact with adults. Fourth, despite having analyzed data from the national surveillance system, the findings described here are probably biased toward symptomatic cases and affected by variations in the testing capacity over time and laboratory errors related to specimen collection, transport, and processing [12,15,20,23,36,46]. Finally, it should be acknowledged that findings were contextualized in a setting where vaccine-induced immunity was virtually absent, and the study of SARS-CoV-2 variants and their properties was poorly understood. Hence, the results described here could be used as a local “baseline” for assessing potential effects on the COVID-19 dynamics after the first 14 months, given the vaccination in groups older than 18 years and SARS-CoV-2 variants.

## 5. Conclusions

Here, using information from the national surveillance system, we provide a comprehensive characterization of the dynamics and epidemiology of COVID-19 among individuals under 18 years in one of the most important departments of the Colombian Caribbean region during the first 14 months of the pandemic. Our findings suggested that the incidence of COVID-19 did not vary by sex but varied by age group. Furthermore, compared to the other municipalities of Bolívar, Cartagena had a higher burden of COVID-19 and experienced early epidemic peaks. Overall, our findings could promote data-driven decision making and inform government policies regarding NPI and vaccines by taking into account potentially distinct dynamics and patterns across population groups and settings.

## Figures and Tables

**Table 1:** Characteristics of children with COVID-19 in Bolívar department and its capital Cartagena from March 2020 to April 2021.

		Confirmed Cases by UNIMOL (N = 1007)	Confirmed Cases by CNIH (N = 7673)	Estimated Population in 2020 ^1^	Cumulative Incidence per 100,000 (95% CI) ^2^
Sex					
	Female	522 (51.8)	3838 (50.1)	361,474	1061.8 (1028.6–1095.7)
	Male	485 (48.2)	3835 (49.9)	343,871	1115.2 (1080.4–1150.9)
Age (Years)					
	p50 (p25–p75)	10 (5–14)	11 (6–14)	—	—
	<1	39 (3.9)	288 (3.8)	39,378	731.4 (649.6–820.5)
	1 to ≤5	193 (19.2)	1111 (14.5)	159,876	649.9 (654.8–736.9)
	5 to ≤10	223 (22.1)	1884 (24.5)	195,887	961.8 (919.0–1006.0)
	10 to ≤14	332 (32.9)	2489 (32.4)	154,577	1610.2 (1548.0–1674.2)
	14 to ≤18	220 (21.9)	1901 (24.8)	155,627	1221.5 (1167.5–1277.3)
Cartagena ^3^					
	No	321 (31.9)	1466 (19.1)	398,915	367.5 (349.0–386.8)
	Yes	686 (68.1)	6207 (80.9)	306,430	2025.6 (1976.0–2076.1)

Note: Data were collected from Laboratorio Unidad de Investigación molecular (UNIMOL) of the Universidad de Cartagena and from the Colombian National Institute of Health (CNIH; https://www.datos.gov.co/Salud-y-Protecci-n-Social/Casos-positivos-de-COVID-19-en-Colombia/gt2j-8ykr/data (accessed on 18 June 2020)). ^1^ The estimated population was obtained from the Departamento Administrativo Nacional de Estadística (DANE) from Colombia. ^2^ The cumulative incidence per 100,000 and its 95% confidence interval were estimated using the number of confirmed cases by the CNIH and the estimated population in 2020. ^3^ Place of residence of SARS-CoV-2-positive children. The “no” category included all municipalities of Bolívar, except Cartagena, which was analyzed independently.

## Data Availability

The data presented in this study are freely available (https://www.datos.gov.co/Salud-y-Protecci-n-Social/Casos-positivos-de-COVID-19-en-Colombia/gt2j-8ykr/data (accessed on 18 June 2021)). Also, the data presented in this study are available on request from the corresponding author.
